# CSE-Induced ER-Mitochondria Crosstalk Promotes Oxidative Stress and Impairs Bronchial Contractile Response

**DOI:** 10.3390/antiox14060703

**Published:** 2025-06-10

**Authors:** Jorge Rodríguez-Pérez, Rosa Andreu-Martínez, Leila Pérez-Sánchez, Ana Hernández-García, Cecilia Muñoz-Calleja, Ángel Cogolludo, María J. Calzada

**Affiliations:** 1 Departamento de Medicina, Facultad de Medicina, Universidad Autónoma de Madrid, 28049 Madrid, Spain; jorge.rodriguezperez@estudiante.uam.es (J.R.-P.); rosa.andreu@estudiante.uam.es (R.A.-M.); leila.perez@uam.es (L.P.-S.); cecilia.munnoz@uam.es (C.M.-C.); 2 Instituto de Investigación Sanitaria Princesa (IIS-Princesa), 28006 Madrid, Spain; 3 Departamento de Farmacología y Toxicología, Facultad de Medicina, Universidad Complutense de Madrid, 28040 Madrid, Spain; anaher10@ucm.es (A.H.-G.); acogolludo@med.ucm.es (Á.C.); 4 Centro de Investigación Biomédica en Red de Enfermedades Infecciosas (CIBERINFEC), Instituto de Salud Carlos III, 28222 Madrid, Spain; 5 Centro de Investigación Biomédica en Red de Enfermedades Respiratorias (CIBERES), Instituto de Salud Carlos III, 28222 Madrid, Spain

**Keywords:** COPD, cigarette smoke, contractility, mitochondria, calcium, ROS

## Abstract

Chronic obstructive pulmonary disease (COPD), whose main risk factor is cigarette smoking, is among the most prevalent diseases worldwide. Previous studies have shown that cigarette smoke extract (CSE) can directly affect pulmonary artery function independently of hypoxia resulting from the airway obstruction. In addition, CSE also affects bronchial smooth muscle, leading to airway hyper-responsiveness. However, its specific impact on the contractile machinery of this compartment remains unclear. In this study, using in vitro experiments with human bronchial smooth muscle cells (hBSMCs), we found that CSE exposure disrupted calcium homeostasis, increased ROS and lipid peroxidation, and reduced cell antioxidant defenses. Furthermore, CSE exposure altered the cell contractile apparatus by decreasing key cytoskeletal proteins and impairing actin dynamics, potentially contributing to the dysregulated contractile response of cells. Notably, these effects were significantly attenuated by antioxidant drugs such as mitoTEMPO and N-acetylcysteine, as well as by the inhibition of the endoplasmic reticulum (ER) calcium channels with 2-aminoethoxydiphenyl borate (2-APB). More importantly, mitoTEMPO partially restored the contractile response of bronchus upon CSE challenge. Collectively, our findings give evidence that CSE-mediated increase in ROS and intracellular calcium contribute to cytoskeletal disruption and functional impairment in airway smooth muscle. Moreover, these results also point to potential therapeutical approaches for mitigating the harmful effects of cigarette smoke in the lung.

## 1. Introduction

Chronic obstructive pulmonary disease (COPD) is one of the most important causes of morbidity, mortality, and healthcare use, affecting 400 million people worldwide [1]. In addition, it is the third leading cause of death globally, being responsible for over 3 million deaths annually [2]. This pulmonary disease develops slowly over many years and noticeable symptoms, such as chronic bronchitis, emphysema, and small airway obstruction, appear several years later. However, the most severe comorbidity of this illness is the development of pulmonary hypertension (PH), due in part to local hypoxia after the emphysema develops [3]. In this context, cigarette smoke (CS) is the main risk factor that contributes to the development and progression of COPD. Nevertheless, environmental factors, such as the rising prevalence of fine particles, dust, or fumes can also play an important role, especially in non-smokers [4].

Bronchodilators and glucocorticoids are the most widely used treatments with therapeutic effects for COPD [5]. However, despite its profound public health impact, curative therapies for COPD or COPD-related pulmonary hypertension are not yet available. Therefore, understanding the molecular mechanisms involved in these pathologies is to develop new therapeutic approaches. In this respect, recent studies in our laboratory have shown that cigarette smoke extract (CSE) has direct effects on the pulmonary vasculature, independently of the hypoxic situation, that lead to a vascular remodeling responsible for the development of pulmonary hypertension [6,7]. On the other hand, studies on the airways demonstrate that CSE promotes airway hyper-responsiveness, an effect that gradually drives into an unusual narrowing of the airways due to smooth muscle contraction [8]. In this context, bronchial smooth muscle cells play a central role in regulating airway function; therefore, it is essential to unveil the mechanisms whereby CSE treatment impairs their performance.

The accumulation of reactive oxygen species (ROS) has been closely associated with COPD pathogenesis, as evidenced by increased oxidative stress markers in exhaled breath condensates and lung tissues of COPD patients compared to control subjects [9]. This phenomenon may be caused not only by oxidizing substances such as tobacco, but also by an excessive production of these species or a decrease in the antioxidant defenses of the cells. In this respect, other authors have identified lower levels of glutathione (GSH) in bronchoalveolar lavages (BALs) from COPD patients [10], and our group has shown that treatment with CSE promotes not only increased oxidative stress, but also a dysregulated antioxidant response in pulmonary arterial smooth muscle cells [7]. Indeed, the excessive ROS production triggers a redox imbalance that contributes to organelle damage and disrupted homeostasis [11]. In particular, mitochondria are considered one of the main sources of ROS in the cell [12], and mitochondrial ROS (mtROS) are routinely produced at several sites within the organelle. However, when a sudden or sustained increase in mtROS exceeds the natural antioxidant defense mechanisms in the cell, the resulting oxidative stress impairs cellular function and viability, ultimately contributing to the onset and progression of COPD [13]. Therefore, elucidating the role of CSE-induced oxidative stress in airway contractile impairment is of particular interest in the context of COPD. To that end, we will first investigate a set of key proteins involved in the contractile machinery of bronchial smooth muscle cells, which may be susceptible to CSE-induced alterations.

Filamentous actin (F-actin) is one of the most representative proteins of the cell cytoskeleton. Specifically, it plays an important role in focal adhesions, alongside other proteins such as vinculin [14]. Furthermore, actin is organized within the cytoplasm of smooth muscle cells where it interacts with myosin to form the core components of the contractile machinery. Both are involved in force generation in a cyclical mechanism that requires myosin light chain (MLC) phosphorylation. However, the contraction of smooth muscle involves many different upstream signals before the latter event occurs. For instance, calmodulin, in the presence of calcium, is known to activate myosin light chain kinase (MLCK), resulting in MLC phosphorylation and contraction [15]. Furthermore, other upstream kinases, such as protein kinase C (PKC), can also contribute to MLCK activation through other signaling pathways [16]. Additionally, the microtubule network also plays a critical role in intracellular dynamics; however, its regulation in smooth muscle cells, particularly in the context of respiratory pathologies such as COPD, remains poorly understood.

In addition to the above-mentioned alterations, other species such as hydroxyl radical and hydroperoxyl [17] can trigger the peroxidation of polyunsaturated fatty acids in biological tissues. Indeed, these products have been shown to display higher levels in breath condensate and in the lungs of stable COPD patients. Moreover, they also correlate negatively with the lung function marker forced expiratory volume in one second (FEV1), suggesting that lipid peroxidation plays an important role in the decline of lung function [18]. Furthermore, it has been shown that this phenomenon also regulates podocyte migration and cytoskeleton structure [19]. Therefore, it is plausible that this phenomenon might also occur within the airway smooth muscle and contribute to the CSE-negative effects in this compartment, such as the dysregulated contractile response.

This study aims to delve deeper into the molecular mechanisms underlying the development of airway hyper-responsiveness induced by cigarette smoke. Our findings demonstrate that cigarette smoke exposure promotes oxidative stress, increases intracellular calcium levels, and disrupts the contractile machinery, collectively contributing to functional impairment of the bronchial smooth muscle layer. Furthermore, we show that several pharmacological agents, particularly antioxidants, effectively mitigate these CSE-induced alterations in the airways.

## 2. Materials and Methods

**Cell culture.** Primary bronchial smooth muscle cells (hBSMCs) were obtained from ScienCell Research Laboratories (#3400), and cultured following the manufacturer’s recommendations in smooth muscle cell medium (ScienCell, #1101, Carlsbad, CA, USA) with 2% fetal bovine serum (ScienCell, #0010), 1× smooth muscle cell growth supplement (ScienCell, #1152), and penicillin/streptomycin solution (ScienCell, #0503). Cells were grown to 90% maximum confluence, maintained in incubators at 37 °C and 5% CO_2_ in a humidified atmosphere, and used for a maximum of eight passages. In the indicated experiments, the cells were pretreated with 25 nM of the mitochondrial antioxidant mitoTEMPO (Sigma, SML0737, St. Louis, CA, USA) for 24 h or with 500 μM N-acetylcysteine (NAC) (Sigma, A7250) for 2 h before being co-treated with 15% CSE. The rest of the treatments were as follows: before CSE treatment, cells were pretreated with 50 μM 2-Aminoethoxydiphenyl borate (2-APB) (Merck Millipore, 100065, Darmstadt, Germany) for 2 h or 5 μM cytochalasin B (Merk, C6762) for 2 h. Afterwards, the cell medium was washed out and replaced with fresh medium containing 15% CSE. When treated with CSE, cells were maintained in a different incubator to avoid the exposure of non-treated cells to volatile substances from the CSE.

**Cigarette smoke extract preparation**. CSE was prepared from commercial Kentucky 3R4F cigarettes (University of Kentucky Lexington, KY, 9.4 mg tar and 0.73 mg nicotine per cigarette). The smoke from the complete burning of five cigarettes was bubbled into 150 mL of phosphate-buffered saline using the VACUSAFE aspiration system (INTEGRA Biosciences AG, Zizers, Switzerland). The CSE solution was then filtered, aliquoted, and kept frozen at −80 °C until use. To ensure a similar preparation amongst different batches, the CSE concentration of the initial solution was measured spectrophotometrically at a 320 nm wavelength. This solution was considered to be 100% CSE, being then diluted to obtain the desired concentration for each experiment.

**Western blot analysis.** Cells were grown to 90% confluence on culture plates in the presence or absence of CSE for different times and lysates were prepared in non-reducing 2X Laemmli buffer, boiled at 95 °C for 10 min in the presence of 20 mM Dithiothreitol (DTT), electrophoretically separated by either an 8% (high MW proteins) or a 15% (low MW proteins) SDS-PAGE gel and transferred onto 0.45 μm nitrocellulose membranes (GE Healthcare Life Sciences, 10600003, Marlborough, MA, USA). Total protein bands were reversibly stained with Fast Green FCF (Sigma, F7252) and imaged on Amersham ImageQuant 800 (GE Healthcare Life Sciences) for total protein quantification. Transferred proteins were incubated overnight at 4 °C with specific primary antibodies (Table 1). Horseradish peroxidase conjugated secondary antibodies (Table 2) were added for 1 h at room temperature and protein signal was then visualized using Immobilon Forte (MerkMillipore, WBLUF0500,) on an Amersham ImageQuant 800. The intensity of specific protein bands was quantified by densitometry using ImageJ 1.51 software (U.S. National Institutes of Health, Bethesda, Maryland, USA) and normalized to the intensity of Fast Green FCF staining from each gel line.

**Cell hypertrophy assessment.** Cells were grown in 35 mm plates in the presence of CSE for 48 h. Afterwards, they were detached with 0.05% trypsin–EDTA (Gibco, 25300-054, NY, USA) and analyzed by flow cytometry. Median forward scattering of light from Ar 488 nm laser was quantified on a FACS Canto™ II cytometer (BD Biosciences, NJ, USA), excluding cell debris and doublets from the analysis.

**Proliferation assay.** Cells were stained with 5 μM fluorescent probe carboxyfluorescein succinimidyl ester (CFSE, CellTraceTM C34554) from Thermo Fisher Scientific (MA, USA) in PBS for 20 min at 37 °C. Afterwards, complete medium was added for another 5 min to stop the staining process and the cells were allowed to attach on 24-well plates before CSE challenge. The cells were detached with 0.05% trypsin–EDTA (Gibco, 25300-054), and median CFSE fluorescence intensity was measured on a FACS CantoTM II cytometer (BD Biosciences), illuminating with an Ar 488 nm laser, right before CSE exposure (day 0 condition) or 72 h later. The results were expressed as the lost CFSE fluorescence with respect to day 0 condition and normalized to CSE-untreated cells’ fluorescence levels.

**Cell senescence assay**. Cell senescence was measured with the Invitrogen™ CellEvent™ Senescence Green Flow Cytometry Assay Kit (C10840, Thermo Fisher Sci. MA, USA). Cells were treated with or without 15% CSE for 72 h. After this time, the cells were trypsinized and washed twice with 1× PBS. Then, the cells were fixed in 4% paraformaldehyde for 10 min at room temperature, and stained with the CellEvent™ Senescence Green Probe diluted 1/1000 in CellEvent™ Senescence Buffer for 2 h at 37 °C. the cells were washed in 1× PBS with 1% BSA, resuspended in 1× PBS, and analyzed by flow cytometry.

**Quantification of calcium, cytosolic, and mitochondrial superoxide levels**. hBSMCs were grown in 35 mm plates and exposed to CSE and/or different drugs. After that, cytosolic and mitochondrial superoxide levels, as well as intracellular calcium levels, were analyzed with fluorescent probes with 10 μM dihydroethidium (DHE) (Invitrogen, D11347), 5 μM MitoSOXTM Red (Thermo Fisher Scientific, M36008), and 1 μM Fluo-4 AM (ThermoFisher Scientific, F14201), respectively. Afterwards, the cells were trypsinized and quantified by flow cytometry. Events were collected on a FACS Canto II flow cytometer. To ensure accuracy and consistency in the flow-cytometry-based quantification of these parameters, the cells were initially selected based on their forward scatter (FSC) and side scatter (SSC) parameters to exclude debris and non-cellular events. Subsequently, doublets were excluded using FSC-H vs. FSC-A analysis, ensuring that only single cells were analyzed. Finally, viable cells were identified by excluding cell labelled positive with ghost dye (Cytekbio, 13-0865-T100, CA, USA), thereby allowing the analysis to focus exclusively on live, single hBSMCs. All collected data were analyzed by GraphPad Prism 9.5.1.

**Quantification of lipid peroxidation.** hBSMCs were grown on 35 mm plates and exposed to CSE and/or different drugs. Afterwards, C11-BODIPY 581/591 staining was conducted. Briefly, a 1.5 mM solution of C11-BODIPY was prepared in DMSO and then diluted in Opti-MEM to achieve a final concentration of 1 μM. The cells were then incubated in the working solution for 30 min at 37 °C, trypsinized, and quantified by flow cytometry. The degree of lipid peroxidation was quantified by calculating the fluorescence intensity ratio between 510 nm (the emission peak of its oxidized form) and 590 nm (the emission peak of its reduced form). Events were collected on a FACS Canto II flow cytometer. To ensure accuracy and consistency in the flow cytometry-based quantification of these parameters, the cells were initially selected based on their forward scatter (FSC) and side scatter (SSC) parameters to exclude debris and non-cellular events. Subsequently, doublets were excluded using FSC-H vs. FSC-A analysis, ensuring that only single cells were analyzed. Finally, viable cells were identified by excluding ghost dye (Cytekbio, 13-0865-T100)-positive events, thereby allowing the analysis to focus exclusively on live, single hBSMCs. All collected data were analyzed by GraphPad Prism 9.5.1.

**Immunofluorescence.** hBSMCs were seeded onto fibronectin (5 μg/mL)-coated 13 mm glass coverslips and then incubated under the desired experimental conditions. The cells were then fixed with 4% paraformaldehyde in PBS for 20 min at 4 °C and permeabilized with 0.3% saponin in 0.2% BSA/PBS. Next, they were labeled with fluorescent primary antibodies (anti-P-MLC and anti-vinculin) followed by Alexa Fluor 488- and 568-labeled secondary antibodies, respectively, and phalloidin-conjugated Alexa 647. A DAPI probe was used to stain the cell nuclei. Cells mounted in coverslips with Faramount Aqueous Medium (Agilent, S3025, CA, USA) were imaged using the THUNDER Imager 3D fluorescence microscope (Leica Microsystems, Wetzlar, GE) and processed with LASX software version 5.1.0. Ten randomly selected images were collected for each sample.

**Cytoskeleton recovery assay.** The actin cytoskeleton was destabilized by incubation for 2 h with 5 μM cytochalasin B (cytB) (Sigma-Aldrich, C6762-1MG, St. Louis, CA, USA) followed by a 1 h recovery period, during which cells were cultured with or without 15% CSE exposure. NAC pretreatment was carried out for 2 h before adding cytB. Then, NAC was withdrawn during destabilization with cytB and reintroduced during the recovery period. Finally, the cells were labeled with Phalloidin Alexa FluorTM 568 (Invitrogen, A12380) at 1:200, trypsinized, and analyzed by flow cytometry. To calculate the percentage of repolymerized F-actin, the MFI of cells with cytoskeletons destabilized by cytB was normalized to the MFI of cells subjected to the same conditions without cytB.

**Bronchial reactivity analysis.** Bronchial segments from 8-week-old C57BL/6J female mice were carefully dissected free of surrounding tissue and cut into rings (1.8–2 mm length). The bronchial rings were then incubated for 24 h in DMEM (Sigma-Aldrich, D5796) medium containing a vehicle (Control) and CSE with or without mitoTEMPO (25 nM). Thereafter, the rings were mounted in a wire myograph (610 M, Danish Myo Technology, Hinnerup, Denmark) with Krebs physiological solution, maintained at 37 °C, and bubbled with a mixture of 21% O**_2_** and 5% CO**_2_**. Contractility was recorded by an isometric force transducer and a displacement device coupled with a digitalization and data acquisition system (PowerLab, ADInstruments, Dunedin, NZ). The rings were stretched to give an equivalent transmural pressure of 20 mmHg. After equilibration, bronchial rings were first stimulated with KCl (80 mM). Then, the preparations were washed three times and allowed to recover before a new stimulation. After that, concentration–response curves for the response to serotonin (5-HT, 30 nM–10 µM) or acetylcholine (ACh 1 nM–10 µM) were created.

**Statistics.** Data are presented as the mean ± SEM. Two-sided unpaired Student’s *t*-tests were used to compare two experimental groups, and one-way or two-way ANOVAs followed by Sidak’s post hoc test were used when comparing three or more groups in accordance with the conditions of normality and homoscedasticity. In case the assumptions of normality and homoscedasticity were not accomplished, the Wilcoxon test was used to compare two groups and the non-parametrical Kruskal–Wallis test was used to compare three or more groups, followed by a Wilcoxon post hoc test to compare between groups. No statistically significant differences were considered to be present when *p* values were larger than 0.05. Statistical and graphical analyses were performed with GraphPad Prism 9.5.1 software (San Diego, CA, USA).

## 3. Results

### 3.1. CSE Induces an Increase in Cytosolic and Mitochondrial ROS, Intracellular Calcium and Lipid Peroxidation in hBSMCs

Given the close association between ROS production and COPD, and the crucial role of calcium in regulating the contractile response, we first analyzed the levels of ROS, specifically the levels of oxygen superoxide, and intracellular calcium in human bronchial smooth muscle cells (hBSMCs) following CSE treatment.

**Figure 1:**
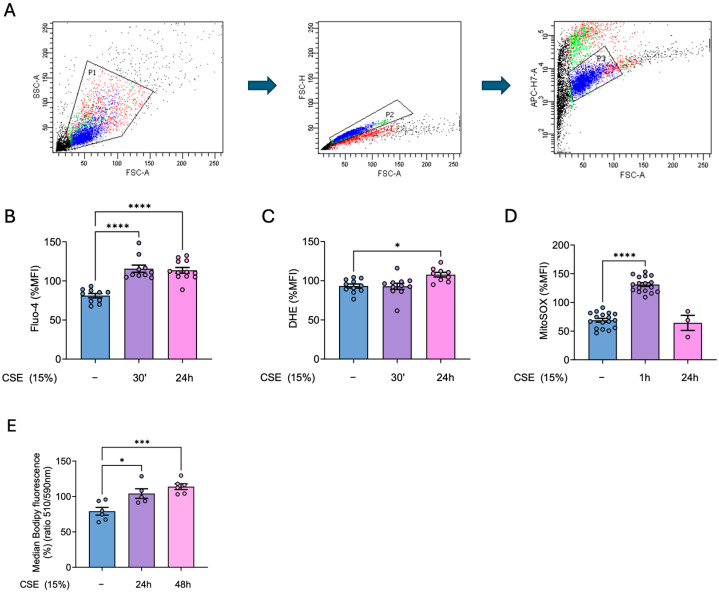
**CSE effects in mitochondrial and cytosolic superoxide levels, intracellular calcium and lipid peroxidation**. Gating strategy to identify the population of interest of hBSMCs (**A**). hBSMCs were exposed to 15% CSE for 30 min or for 1, 24, or 48 h; afterwards, flow cytometry analysis was performed. Cells were stained with 1 µM Fluo-4AM (**B**) and 10 µM DHE (**C**) to analyze calcium and cytosolic superoxide levels, respectively, or with 5 µM MitoSOX Red to analyze mitochondrial superoxide (**D**). Lipid peroxidation was analyzed with 1 µM Bodipy 581/591 C11 and data were calculated as the ratio between the oxidized (510 nm) and the non-oxidized (590 nm) form (**E**). Results are presented as mean ± SEM, n = 3–17. Statistical analysis between three or more groups was carried out using one-way ANOVA followed by Sidak’s post hoc test. * *p* < 0.05, *** *p* < 0.001, **** *p* < 0.0001.

We first established a standardized gating strategy to identify the population of interest, excluding debris, non-cellular events, and doublets. Furthermore, viable cells were identified by excluding ghost-dye-positive events (APCH7-A positive), thereby allowing the analysis to focus exclusively on live, single hBSMCs (Figure 1A). Our results showed that CSE promoted a rapid and significant increase in intracellular Ca^2+^ levels, peaking at 30 min post-exposure (Figure 1B). Moreover, this increase was accompanied by a small but significant increase in cytosolic superoxide levels, reaching its maximum at 24 h after CSE treatment (Figure 1C). In addition, the superoxide levels generated by the mitochondria were significantly higher in CSE-treated cells with a quick increase peaking 1 h after CSE exposure (Figure 1D). This increase in ROS likely contributed to a marked increase in lipid peroxidation in hBSMCs upon 24 h of CSE exposure, with levels peaking at 48 h post-exposure (Figure 1E). This oxidative damage may play a key role in mediating the harmful effects of CSE in this compartment.

### 3.2. CSE Stimulates ER–Mitochondria Crosstalk, Triggering a Feedback Loop That Amplifies Both ROS and Calcium Levels

**Figure 2:**
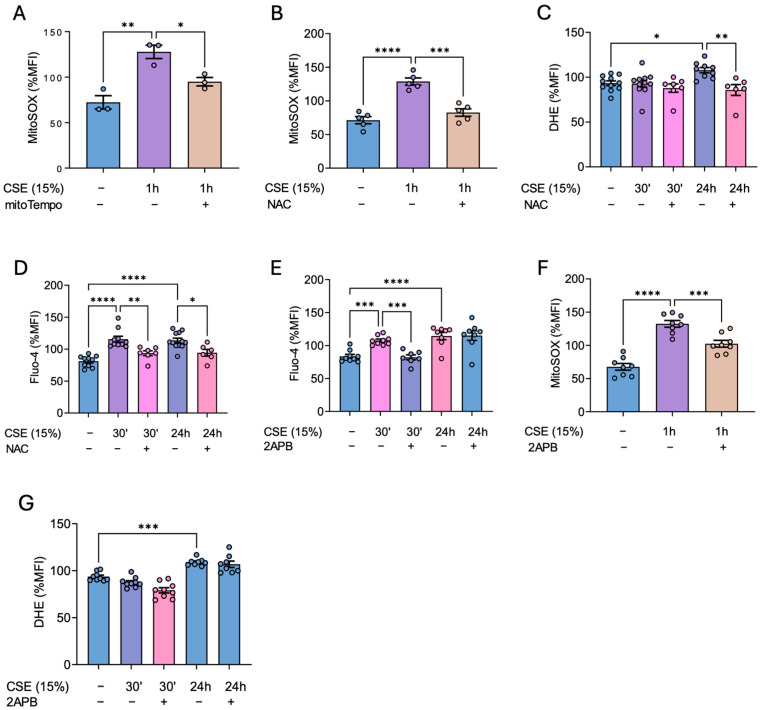
**Effects of antioxidants and ER calcium channel inhibition on CSE-mediated increase in intracellular calcium and ROS.** Cells were cultured for 2 h in the absence (−) or the presence of 500 µM NAC and 25 nM mitoTEMPO before adding 15% CSE for the indicated time. Regarding 2-APB, cells were pretreated with 50 µM of the drug for 4 h. Afterwards, 2APB was removed and the cells were exposed to 15% CSE for the indicated time. After that, the cells were stained with 5 µM MitoSOX Red to analyze mitochondrial superoxide (**A**,**B**) or probed with 10 µM DHE (**C**) and 1 µM Fluo-4AM (**D**) to analyze cytosolic ROS and calcium levels, respectively, via flow cytometry. The effect of 2-APB on the CSE-mediated increase in calcium (**E**)**,** mitochondrial (**F**), and cytosolic (**G**) superoxide were analyzed at the indicated times. The results are presented as mean ± SEM, n = 3–10. Statistical analysis between three or more groups was carried out using one-way ANOVA followed by Sidak’s post hoc test. * *p* < 0.05, ** *p* < 0.01, *** *p* < 0.001, **** *p* < 0.0001.

Given that CSE treatment induced a rise in superoxide in hBSMCs, we sought to explore the underlying regulatory mechanisms and potential strategies to prevent it. First, we analyzed whether antioxidants could prevent the CSE-mediated alterations in ROS homeostasis. As expected, our results showed that pretreatment with mitoTEMPO (a mitochondria-targeted antioxidant) could prevent the increase in mitochondrial superoxide stimulated by CSE treatment (Figure 2A). Moreover, pretreatment with NAC not only prevented the increase in mitochondrial and cytosolic superoxide levels but also inhibited the rise in intracellular calcium (Figure 2B–D).

These results suggested that tobacco may induce a bidirectional regulation of calcium and ROS. To explore this, we next assessed the effect of inhibiting calcium channels from the endoplasmic reticulum as inositol 1,4,5-triphosphate receptor (IP3R). Specifically, we evaluated the efficacy of 2-Aminoethoxydiphenilborate (2-APB), a membrane-permeable IP3R inhibitor, in preventing the increase in calcium and ROS.

Our results showed that inhibiting the endoplasmic reticulum IP3R with 2-APB prevented the initial rise in intracellular calcium (Figure 2E) and, more importantly, it also prevented the increase in mitochondrial superoxide (Figure 2F). However, contrary to our expectations, the late increase in cytosolic ROS observed 24 h after CSE treatment could not be prevented with this drug (Figure 2G). These results therefore suggested the involvement of an ER–mitochondrial axis involved in the CSE-mediated increase in mtROS and calcium in hBSMCs.

### 3.3. CSE Decreases Antioxidant Defenses in hBSMC

**Figure 3:**
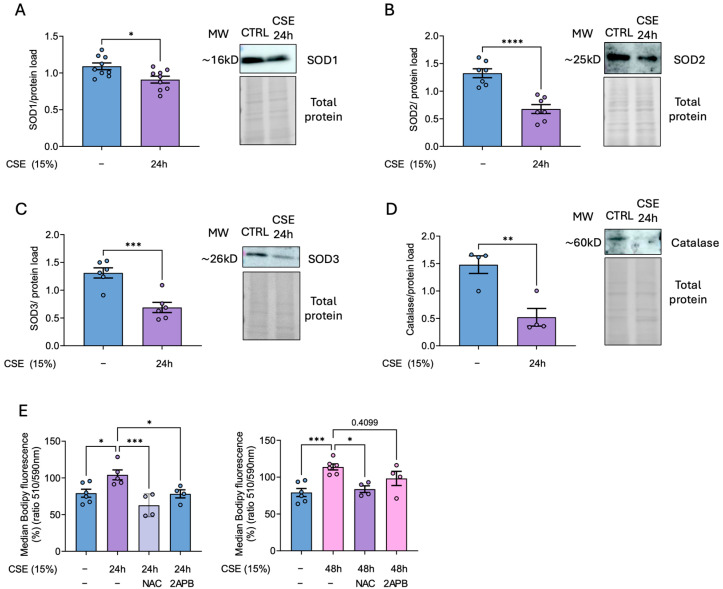
**Analysis of antioxidant enzyme proteins levels and lipid peroxidation in CSE-treated hBSMCs.** hBSMCs were exposed to 15% CSE for 24 h and SOD1, SOD2, SOD3 and catalase protein levels were analyzed by Western blot (**A**–**D**). Cells were exposed to 15% CSE in the absence (−) or presence of 500 µM NAC or pretreated for 4 h with 2-APB before CSE treatment. Cells were then probed with 1 µM Bodipy 581/591 C11 and lipid peroxidation data were calculated as the ratio between the oxidized (510 nm) and the non-oxidized (590 nm) form (**E**). The results are presented as mean ± SEM, n = 4–9. Statistical analysis between three or more groups was carried out using one-way ANOVA followed by Sidak’s post hoc test. Statistical analysis between two groups was carried out using a two-sided unpaired Student’s *t*-test. * *p* < 0.05, ** *p* < 0.01, *** *p* < 0.001, **** *p* < 0.0001.

Our previously published results demonstrate that CSE impairs the antioxidant response in pulmonary artery smooth muscle cells [7]. Given the significant increase in mitochondrial superoxide in CSE-treated hBSMCs, we examined whether a reduction in certain cellular antioxidant enzymes contributed to this rise. Our results supported this hypothesis, showing that CSE treatment significantly reduced antioxidant defenses, specifically the levels in superoxide dismutase isoforms SOD1, SOD2, and SOD3, with the most pronounced decrease observed in the mitochondrial (SOD2) and extracellular (SOD3) forms (Figure 3A–C). In addition, catalase levels were also markedly diminished upon CSE exposure (Figure 3D). The resulting accumulation of superoxide, reinforced by impaired antioxidant defenses, likely contributed to the observed increase in lipid peroxidation, as previously shown. More importantly, this effect was totally prevented by treatment with either NAC or 2-APB after 24 h of CSE exposure. In addition, NAC, unlike 2-APB, significantly prevented the increase in lipid peroxidation reported after 48 h of CSE (Figure 3E).

### 3.4. hBSMC Exhibits a Senescent Phenotype in Response to CSE

**Figure 4:**
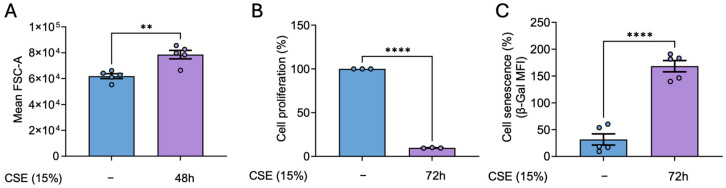
**Effects of CSE in cell size, proliferation, and senescence of hBSMCs**. Cells were untreated (−) or exposed to 15% CSE for 48 h and cell size was quantified in terms of the FSC-A parameter using flow cytometry (**A**). For proliferation assays, cells were stained with 5 µM of CFSE, and median fluorescent intensity was measured by flow cytometry right before the CSE exposure (day 0 condition) or after 72 h in the presence or the absence of 15% CSE. Data are presented as the lost CFSE fluorescence with respect to day 0 condition and normalized to CSE-untreated cells’ fluorescence levels (**B**). X-gal staining to quantify β-Galactosidase activity was analyzed by flow cytometry. Cell senescence is presented as median fold change in β-Gal MFI and normalized to the levels in non-CSE-treated (-) cells (**C**). n = 3–5. Statistical analysis between two groups was carried out using a two-sided unpaired Student’s *t*-test. ** *p* < 0.01 **** *p* < 0.0001.

Our previous findings demonstrate that CSE induces cell senescence in hPASMCs [6]. Thus, we studied whether the molecular alterations observed in response to CSE in hBSMCs were also associated with the induction of a senescence phenotype. To this end, we assessed established markers of senescence, including changes in cell size and proliferative capacity. Our results proved that CSE treatment resulted in a significant increase in cell size as well as a marked reduction in proliferation (Figure 4A,B). These changes were consistent with the emergence of a senescent phenotype, which became evident 72 h after CSE exposure (Figure 4C).

### 3.5. CSE Decreases the Levels of Proteins Involved in Cytoskeleton Dynamics in hBSMCs

**Figure 5:**
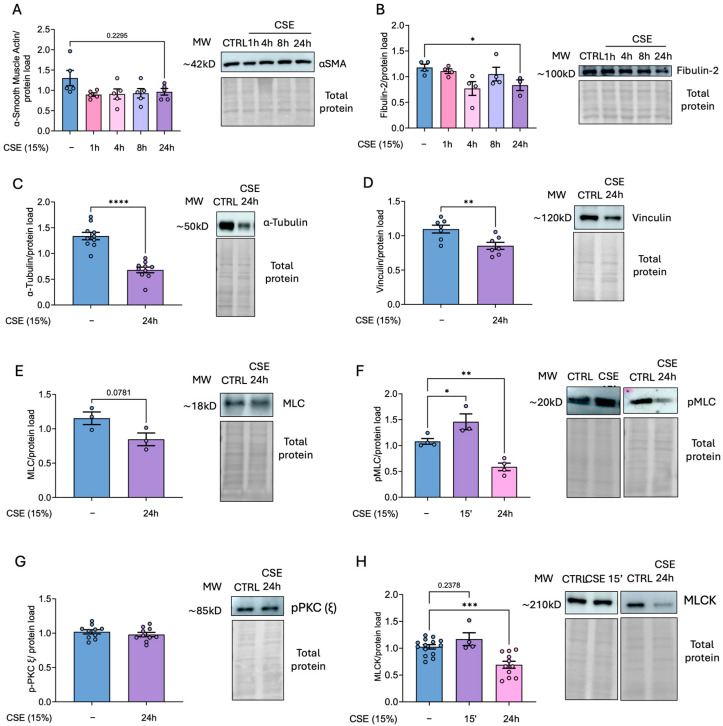
**Effects of CSE on representative proteins of cell cytoskeleton in hBSMCs**. Protein levels from hBSMCs cultured in the presence of 15% CSE for different times were determined by Western blot using primary antibodies for the indicated proteins (**A**–**H**). Total protein was used as loading control. Representative images and band quantifications by densitometry are shown. Protein levels are presented as mean ± SEM, n = 3–18. Statistical analysis between three or more groups was carried out using one-way ANOVA followed by Sidak’s post hoc test. Statistical analysis between two groups was carried out using a two-sided unpaired Student’s *t*-test. * *p* < 0.05, ** *p* < 0.01, *** *p* < 0.001, **** *p* < 0.0001.

As previously noted, airway hyper-reactivity is a common feature in COPD patients, and hBSMCs play a pivotal role in mediating this response. Since cell traction machinery is crucial to this process, we investigated whether CSE might affect the levels of some representative cytoskeletal proteins. To this end, we challenged hBSMCs to 15% CSE for varying durations to assess time-dependent effects and then analyzed protein levels using Western blots. Although the levels of α-smooth muscle actin, which is a major component of the cytoskeleton and plays a central role in cell structure, remained unchanged, other cytoskeletal proteins including fibulin and tubulin were significantly diminished following 24 h of CSE treatment (Figure 5A–C). Additionally, we analyzed the impact of smoke exposure on proteins of focal adhesions, since these are dynamic protein complexes playing a crucial role in mechanotransduction. Similarly, the levels of vinculin were significantly decreased in CSE-treated cells (Figure 5D). Next, we analyzed the levels of MLC and its phosphorylated active form p-MLC. Our results showed no significant changes in total MLC levels after 24 h of CSE exposure. However, phosphorylated, active MLC levels increased at 15 min post-exposure, only to decrease significantly after 24 h (Figure 5E,F). To address whether the effects on MLC phosphorylation were due to alterations in specific kinases, we analyzed the levels of MLCK (responsible for the phosphorylation and activation of MLC), and the levels of pPKC (ε) as an upstream effector of this signaling pathway. Although the levels of pPKC (ε) remained unchanged, MLCK levels showed only a slight (but not significant, *p* = 0.2378) increase 15 min after CSE exposure and was followed by a significant reduction after 24 h of treatment (Figure 5 G,H).

### 3.6. CSE Disrupts Actin Cytoskeleton Assembly and Impairs Its Spontaneous Recovery in hBSMCs After Cytochalasin B Treatment

**Figure 6:**
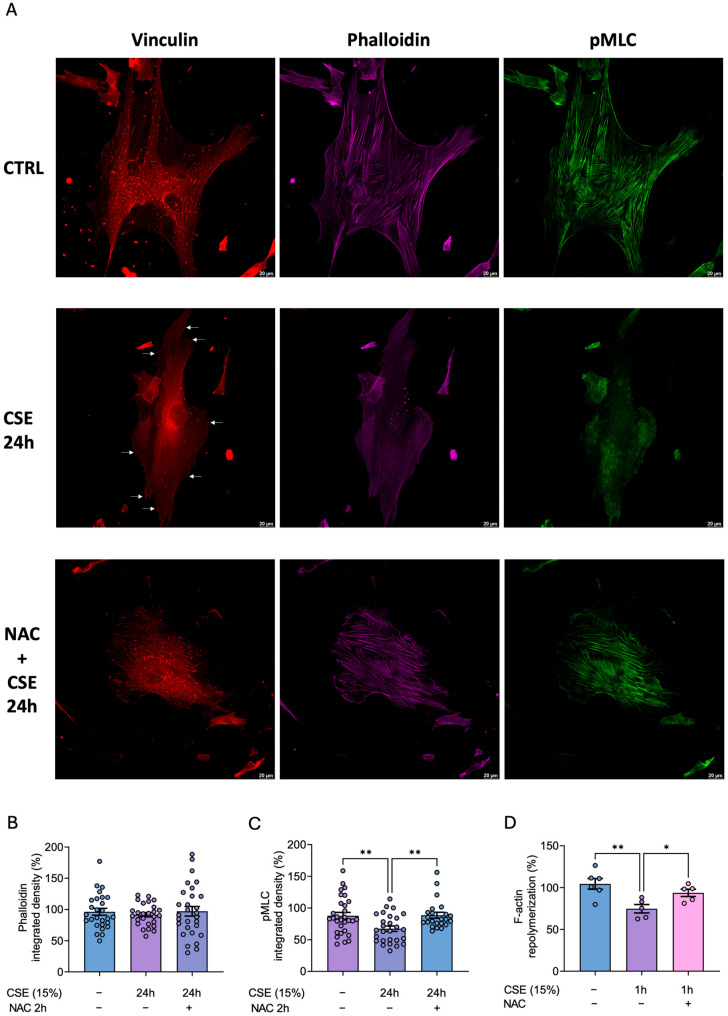
**Effects of CSE in cytoskeleton dynamics**. hBSMCs were cultured in the absence or presence of 15% CSE for 24 h and then stained for pMLC (green), F-actin (magenta), or vinculin (red). Then, 8–10 different fields were analyzed and representative images from each sample were taken with a Thunder imager (Leica microsystems), n = 3; with white arrows pointing to vinculin-positive focal adhesions (**A**). Phalloidin and pMLC fluorescent intensity was quantified by means of Integrated Density using ImageJ software (**B**,**C**). hBSMCs were treated for 2 h with 5 µM cytochalasin B and then cultured for 1 h without the drug (control condition) or in the presence of 15% CSE. Cells were also pretreated with 500 µM NAC for 2 h before adding cytochalasin B for another 2 h. Finally, they were cultured for 1 h in the presence of NAC and 15% CSE (**D**)**.** Statistical analysis between two groups was carried out using a two-sided unpaired Student’s *t*-test. Statistical analysis between three or more groups was carried out using one-way ANOVA followed by Sidak’s post hoc test. * *p* < 0.05, ** *p* < 0.01.

To gain a better understanding not only of the levels of these proteins but also of their assembly within the cell cytoplasm, we performed immunofluorescence microscopy (Figure 6A). In agreement with our Western blot data, CSE decreased vinculin levels and changed its subcellular location from the cytoplasm to the cell periphery; these effects were partially prevented with NAC (Figure 6A). Although actin filaments appeared diffuse and poorly organized in the CSE-treated cells, their quantitative levels remained unchanged (Figure 6A,B). In addition, the p-MLC fluorescence intensity was significantly reduced in the CSE-treated cells, and this effect was also significantly prevented with NAC (Figure 6C).

To reconcile the discrepancy between the levels of actin and its organization, we performed an actin dynamics assay to assess cytoskeleton recovery capability following disruption by cytochalasin B. This drug is a cell-permeable mycotoxin that inhibits actin polymerization into filamentous actin. Specifically, it blocks actin monomer addition to the “barbed” end of the filaments, where monomers are normally added more rapidly [20]. Thus, hBSMCs were treated with cytochalasin B for 2 h, followed by a 1 h recovery period in drug-free medium, with or without CSE. Cytoskeletal organization was then evaluated using phalloidin staining and analyzed by flow cytometry. Our results showed that CSE-treated cells exhibited a significantly reduced actin repolymerization rate compared to controls (Figure 6D). Most importantly, pretreatment with the antioxidant NAC efficiently prevented this phenotype (Figure 6D). Therefore, these results indicated that the CSE-mediated increase in ROS is responsible for the alterations in the cytoskeleton.

### 3.7. MitoTEMPO Alleviates CSE-Mediated Decrease in Bronchial Contractility

**Figure 7:**
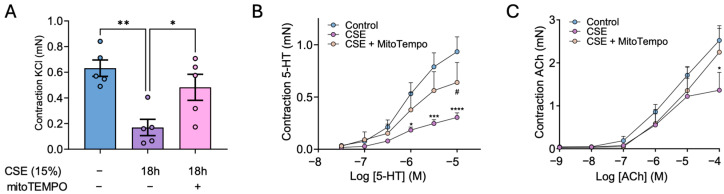
**Effects of CSE in bronchial reactivity.** Mice bronchial rings were incubated with vehicle (Control) and 15% CSE with or without 25 nM mitoTEMPO. Contractile responses induced by 80 mM KCl (**A**), 5-HT (**B**), and ACh (**C**) were then quantified with a wire myograph. Data are presented as mean ± SEM; n = 6–7 mice for A and B and n = 4–6 mice for C. Statistical comparisons among groups were made using one-way (A) or repeated-measures two-way (B and C) ANOVA followed by Bonferroni’s post hoc test (* *p* < 0.05, ** *p* < 0.01, *** *p* < 0.001, **** *p* < 0.0001 vs. Control; # *p* < 0.05 vs. CSE).

Since CSE exposure disrupted the expression of key proteins involved in smooth muscle contractility, we further tested whether it might alter bronchial reactivity. To this end, mice bronchial rings were incubated with vehicle (Control) or CSE with or without mitoTEMPO and mounted in a wire myograph. We found that incubation with CSE markedly reduced the bronchial contraction to KCl (Figure 7A), to 5-HT (Figure 7B) and, to a lesser extent, to ACh (Figure 7C). Interestingly, mitoTEMPO alleviated the CSE-induced decrease in bronchial contraction to Ach, and, more importantly, significantly prevented the decrease in contraction to KCl and 5-HT.

## 4. Discussion

COPD is a complex respiratory pathology characterized by chronic bronchitis, emphysema, small airway obstruction, and persistent respiratory symptoms. It is well known that cigarette smoke, which is the main risk factor of this illness, is key to promoting airway hyper-responsiveness. However, the mechanisms involved in CSE-mediated effects on airway smooth muscle are poorly understood. Here, we have delved into the molecular mechanisms responsible for the generation of ROS and the deregulation of calcium levels in bronchial smooth muscle cells following exposure to tobacco smoke, and how these pathways contribute to the smoke-induced dysregulation in airway hyperreactivity.

We documented two different peaks of calcium occurring at 30′ and 24 h after CSE exposure. This suggests that, despite calcium signaling occurring rapidly after the initial stimuli, this signal could be extended for longer periods of time due factors such as the continuous presence of CSE in the cell culture or the activation of “long-lasting” calcium channels. This could explain the increase in calcium observed after 24 h of CSE exposure. Furthermore, this second wave of calcium may be amplified by the increase in ROS levels at later times. This is supported by previous results reporting a feedback regulatory mechanism between calcium and ROS [21]. In parallel, antioxidant enzymes such as SOD1, SOD2, SOD3, and catalase were decreased upon CSE treatment, indicating that the rise in oxidative stress is not only due to the direct effects of tobacco smoke, but also to impaired antioxidant defenses in bronchial smooth muscle cells. These findings are in line with previous results from our group regarding pulmonary artery smooth muscle cells [7]. Notably, N-acetylcysteine not only prevented the increase in ROS observed 24 h post CSE exposure, but also blocked the calcium peaks to both 30 min and 24 h after CSE. This result supports the existence of a feedback loop between calcium and ROS and suggests that the early calcium influx in the cells may contribute to the late ROS surge. Additionally, CSE treatment increased mitochondrial superoxide levels, which was also prevented by NAC, further highlighting the effectiveness of this antioxidant in our cells. On the other hand, the reduction in antioxidant defenses and the resulting accumulation of hydrogen peroxide (H₂O₂), due to elevated cytosolic superoxide, likely accounts for the observed increase in lipid peroxidation after 24 h of CSE exposure. Consistent with these findings, NAC effectively prevented this rise in lipid peroxidation, including the peak levels detected at 48 h.

IP_3_ receptors, which mediate calcium release from intracellular stores, are mainly located in the endoplasmic reticulum [22]. In this regard, 2-APB, an antagonist of these receptors, was shown to block the early CSE-driven increase in intracellular calcium as well as the subsequent increase in mitochondrial superoxide. Thus, these results demonstrate that, in response to CSE, an initial cytosolic calcium surge originating from the ER triggers a subsequent overproduction of mitochondrial ROS. This phenomenon may be attributed to mitochondrial calcium uptake through calcium-permeable channels within this organelle, such as voltage-dependent anion channels (VDACs) located in the mitochondrial outer membrane [23]. This situation will, in turn, lead to a mitochondrial calcium overload responsible for the generation of mitochondrial oxidative stress [24].

CSE-mediated oxidative stress and calcium alterations in hBSMCs may be potentially linked to the impaired cytoskeleton function and organization observed in our cells, as some of these effects were prevented with antioxidants. In this context, previous studies have shown that nicotine [25], tobacco smoke exposure [26], and particularly elevated ROS levels [27,28] significantly impact cytoskeletal architecture, focal adhesion dynamics, and the organization of actin and tubulin networks. We also detected altered actin dynamics and the decreased phosphorylation of myosin light chain (MLC), both critical markers of impaired contractile function. These alterations appear to be closely linked to CSE-induced oxidative stress, as they were mitigated by treatment with the antioxidant N-acetylcysteine (NAC). Supporting this, previous research suggests that MLCK activity is modulated by the cellular redox state, with elevated oxidative conditions potentially inhibiting its function, thus reducing MLC phosphorylation [28]. This mechanism may underlie the diminished reactivity of CSE-challenged bronchi observed in our study.

However, these findings are somewhat unexpected in this cellular compartment, as previous studies have reported that CSE exposure leads to increased airway hyperreactivity, primarily driven by enhanced contraction of airway smooth muscle cells [8]. This could be explained by the fact that phosphorylation is a rapid post-translational modification that occurs within minutes in response to specific stimuli. In fact, the analysis of MLC phosphorylation shortly after CSE exposure demonstrated an early increase in pMLC levels, accompanied by a slight but concurrent rise in MLCK, the kinase responsible for MLC phosphorylation. These findings, along with the quick increase in cytosolic calcium, suggest that hBSMCs exhibit an enhanced but short-lived contractile response immediately following CSE exposure. Previous findings from the literature indicate that while chronic exposure to cigarette smoke generally enhances airway contractility, short-term or acute exposure can transiently reduce airway tone, particularly through nicotine-mediated pathways [29,30,31]. A plausible explanation for the reduced pMLC levels observed after 24 h is that CSE may impair global protein synthesis in hBSMCs, which could also account for the decreased levels of other cytoskeleton proteins. In addition, oxidative stress may contribute to protein degradation via oxidative modifications [32]. Notably, treatment with the antioxidant NAC prevented the CSE-mediated loss of cytoskeleton recovery ability and preserved the organization and/or the levels of key cytoskeletal proteins including vinculin, pMLC, and F-actin. These findings indicate that the dysregulation of ROS and calcium homeostasis underlies the observed alterations in cytoskeletal integrity and contractile machinery. Additionally, increased cell senescence in response to CSE point to a broader cellular stress response that compromises both the structural integrity and proliferative capacity of hBSMCs. However, to further characterize this phenotype, future analyses of specific senescence markers such as p53 and p21 would be valuable.

Along with the disrupted expression and organization of crucial cytoskeleton proteins, we also confirmed that CSE reduced bronchial contraction in response to KCl (used as a positive control), 5-HT, and ACh. In this regard, mitoTEMPO alleviated the loss of contractility in response to ACh, and, more importantly, it significantly prevented the CSE-mediated decrease in contractility with KCl and 5-HT. Therefore, these results further reinforce the importance of the early CSE-mediated increase in calcium and ROS in the contractile machinery dysfunction of hBSMCs.

Nevertheless, this study has also certain limitations that should be acknowledged. First, 2-APB may exert additional effects on other calcium channels, such as members of the TRPC family. In this regard, we also performed experiments using verapamil, a calcium channel blocker that primarily inhibits L-type calcium channels on the plasma membrane. Unlike the effects observed with 2-APB on calcium and ROS levels, verapamil treatment did not produce significant changes regarding both of them (Supplementary Figure S1), therefore reinforcing the specificity of 2-APB towards IP3R. On the other hand, our functional experiments were conducted using bronchial rings from female mice only, precluding the assessment of potential sex-based differences in CSE responses or antioxidant efficacy, which requires further research. In addition, although the combination of in vitro and ex vivo models provides valuable mechanistic insight, the extrapolation of these findings to the in vivo context is limited. Long-term in vivo studies using chronic cigarette smoke exposure models would be essential to validate the physiological relevance of the observed alterations.

In conclusion, our results demonstrate that CSE exposure disrupts cytoskeleton organization and function in hBSMCs. This disruption is accompanied by increased levels of superoxide, intracellular calcium, and lipid peroxidation, factors that likely contribute to the detrimental effects of CSE on the contractile apparatus. This hypothesis is further supported by our observation that both antioxidants and calcium-modulating agents effectively mitigate these alterations. Therefore, the use of these drugs, in combination with current therapies, could be a promising therapeutic approach for the management of COPD.

## Figures and Tables

**Table 1:** List of primary antibodies used for western blot (WB), immunofluorescence (IF) and flow cytometry (FC), with the corresponding dilution and product information.

Target Protein	Technique	Clonality (Clone)	Host Species	Dilution	Manufacturer (#Catalog)
**pPKC ε (Ser-729)**	WB	Polyclonal	Rabbit	1:1000	Santa Cruz (sc-12355-R)
**Myosin light chain kinase (MLCK)**	WB	Monoclonal	Rabbit	1:1000	Abcam (ab314185)
**Myosin light chain (MLC)**	WB	Polyclonal	Rabbit	1:1000	Cell Signaling (3672S)
**p-MLC (Thr18/Ser19)**	WB and IF	Polyclonal	Rabbit	1:1000	Cell Signaling (3674)
**Alpha-tubulin**	WB	Monoclonal	Mouse	1:1000	Sigma (T9026)
**Alpha smooth muscle actin**	WB	Monoclonal	Rabbit	1:2000	Abcam (ab124964)
**Fibulin-2**	WB	Polyclonal	Rabbit	1:1000	Abcam (Ab234993)
**Cofilin-1**	WB	Monoclonal	Mouse	1:1000	Abcam (Ab54532)
**SOD1**	WB	Monoclonal	Mouse	1:200	Santa Cruz (sc-101523)
**SOD2**	WB	Monoclonal	Mouse	1:1000	Abcam (ab74231)
**SOD3**	WB	Monoclonal	Mouse	1:1000	Abcam (Ab80946)
**Catalase**	WB	Monoclonal	Mouse	1:200	Santa Cruz (sc-271358)
**Vinculin**	WB and IF	Monoclonal	Mouse	1:2000	Sigma-Aldrich (V9131)
**Phalloidin (Alexa Fluor^TM^ 647)**	IF	-	-	1:1600	Invitrogen (A22287)
**Phalloidin (Alexa Fluor^TM^ 568)**	FC	-	-	1:200	Invitrogen (A12380)

**Table 2:** List of primary antibodies used for western blot (WB) and immunofluorescence (IF) with the corresponding dilution and product information.

Target Protein	Conjugated	Dilution	Manufacturer (#Catalog)
**Goat anti-rabbit IgG**	HRP	1:2000	Invitrogen (A32460)
**Goat anti-mouse IgG**	HRP	1:2000	Dako (P0447)
**Rabbit anti-rat IgG**	HRP	1:2000	Sigma-Aldrich (AP136P)
**Goat anti-rabbit IgG**	Alexa Fluor^TM^ 488	1:100	Invitrogen (A11034)
**Goat anti-mouse IgG**	Alexa Fluor^TM^ 568	1:100	Invitrogen (A11031)

## Data Availability

All the data and materials involved in this study are present in the paper or the Supplementary Materials. Additional information can be provided upon request to the corresponding author.

## Supplementary Materials

The following supporting information can be downloaded at https://www.mdpi.com/article/10.3390/antiox14060703/s1: Figure S1: Effects of verapamil on the CSE-mediated increase in intracellular calcium and cytosolic superoxide. hBSMCs were exposed to 15% CSE for 30 min or 24 h and then flow cytometry analysis was performed. Cells were stained with 1 µM Fluo-4AM (A) and 10 µM DHE (B) to analyze calcium and cytosolic superoxide levels, respectively. Results are presented as mean ± SEM, n = 3–6. Statistical analysis between three or more groups was carried out using one-way ANOVA followed by Sidak’s post hoc test. * *p* < 0.05, ** *p* < 0.01.

## Abbreviations

The following abbreviations are used in this manuscript:

2-APB	2-Aminoethoxidiphenylborate
ACh	Acetylcholine
BAL	Bronchoalveolar lavage
CFSE	Carboxyfluorescein succinimidyl ester
COPD	Chronic obstructive pulmonary disease
CSE	Cigarette smoke extract
CytB	Cytochalasin B
DHE	Dihydroethidium
DMEM	Dulbecco’s Modified Eagle Medium
ER	Endoplasmic reticulum
FACS	Fluorescence-Activated Cell Sorting
FBS	Fetal Bovine Serum
FSC	Forward Scatter
FSC-A	Forward Scatter Area
FSC-H	Forward Scatter Height
FEV1	Forced expiratory volume in one second
GSH	Glutathione
hBSMC	Human bronchial smooth muscle cells
IP3R	Inositol 1,4,5-triphosphate receptor
MLC	Myosin light chain
MLCK	Myosin light chain kinase
mtROS	Mitochondrial ROS
NAC	N-acetylcysteine
PH	Pulmonary hypertension
PKC	Protein kinase C
ROS	Reactive oxygen species
SEM	Standard Error of the Mean
SOD	Superoxide dismutase
SSC	Side Scatter
VDAC	Voltage-Dependent Anion Channel
