# Supplementary material for: CSE-Induced ER-Mitochondria Crosstalk Promotes Oxidative Stress and Impairs Bronchial Contractile Response
Source: Antioxidants (Basel). 2025 Jun 10;14(6):703. doi: 10.3390/antiox14060703 (PMC12189939; doi:10.3390/antiox14060703)
Supplement: Supplementary file 1 [file antioxidants-14-00703-s001.zip › antioxidants-3663010-supplementary.pdf]

Supplementary figures

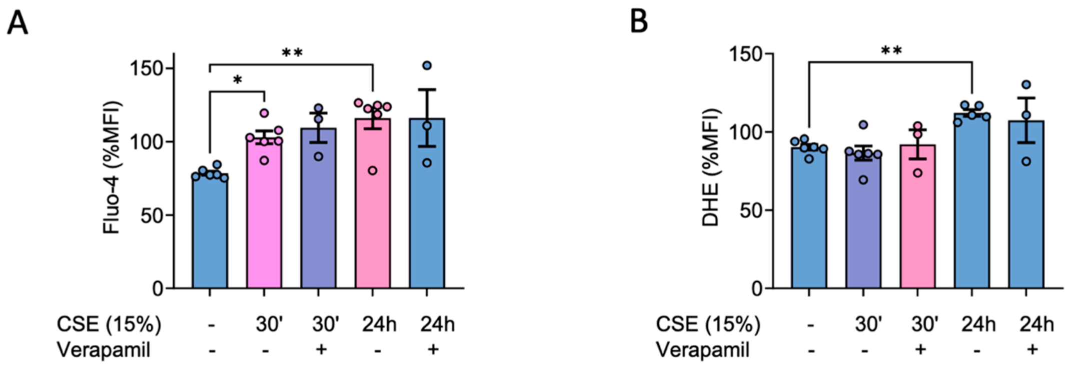

Figure S1. Effects of Verapamil on the CSE-mediated increase in intracellular calcium and cytosolic superoxide. hBSMCs were exposed to 15% CSE for 30 min or 24 h and then flow cytometry analysis was performed. Cells were stained with 1 μM Fluo-4AM (A) and 10 μM DHE (B) to analyze calcium and cytosolic superoxide levels, respectively. The results are presented as mean ± SEM, n = 3-6. A statistical analysis between three or more groups was carried out using one-way ANOVA followed by Sidak's post hoc test. *$p < 0.05$, **$p < 0.01$.
